# Bipolar Disorder and Comorbid Borderline Personality Disorder: Patient Characteristics and Outcomes in US Hospitals

**DOI:** 10.3390/medicina55010013

**Published:** 2019-01-14

**Authors:** Rikinkumar S. Patel, Geetha Manikkara, Amit Chopra

**Affiliations:** 1 Department of Psychiatry, Griffin Memorial Hospital, 900 E Main St, Norman, OK 73071, USA; 2 Department of Psychiatry, Texas Tech University Health Science Center, Midland, TX 79701, USA; geethamanikkara@gmail.com; 3 Department of Psychiatry, Allegheny Health Network, 4 Allegheny Center 8th Floor, Pittsburgh, PA 15212, USA; amit.chopra@ahn.org

**Keywords:** bipolar disorder, borderline personality disorder, personality disorder, comorbidity, inpatient psychiatry, suicidal, management, hospital outcomes, mood disorder, manic episode

## Abstract

*Background and objectives*: The quality of life and disease outcomes in bipolar patients, including increased risk of psychiatric hospitalizations and suicide, are adversely affected by the presence of borderline personality disorder (BPD). Our study aims to determine the impact of BPD on the inpatient outcomes of bipolar disorder patients. *Methods*: We used Nationwide Inpatient Sample from the US hospitals and identified cases with bipolar disorder and comorbid BPD (N = 268,232) and controls with bipolar disorder only (N = 242,379), using the International Classification of Diseases, 9th Revision, and Clinical Modification codes. We used multinomial logistic regression to generate odds ratios (OR) and evaluate inpatient outcomes. *Results*: The majority of the bipolar patients with BPD were female (84.2%), Caucasian (83.1%) and 18–35 years age (53.9%). Significantly longer inpatient stays, higher inpatient charges, and higher prevalence of drug abuse were noted in bipolar patients with BPD. The suicide risk was higher in bipolar patients with BPD (OR = 1.418; 95% CI 1.384–1.454; *p* < 0.001). In addition, utilization of electroconvulsive treatment (ECT) was higher in bipolar patients with comorbid BPD (OR = 1.442; 95% CI 1.373–1.515; *p* < 0.001). *Conclusions*: The presence of comorbid BPD in bipolar disorder is associated with higher acute inpatient care due to a longer inpatient stay and higher cost during hospitalization, and higher suicide risk, and utilization of ECT. Further studies in the inpatient setting are warranted to develop effective clinical strategies for optimal outcomes and reduction of suicide risk in bipolar patients with BPD.

## 1. Introduction

Bipolar disorder is a psychiatric illness characterized by extreme highs and lows in mood and energy [1]. Patients with bipolar disorder experience disability, functional decline, reduction in quality of life, mortality from comorbid medical conditions, and increased service utilization [2]. The lifetime prevalence of bipolar disorder in the US was 2.4%, and point-prevalence was found to be 1.6% [3].

Borderline personality disorder (BPD) is a severe mental illness characterized by unstable mood, interpersonal relationships, self-image, and behavior. This instability often affects family and professional life, and an individual’s sense of identity [4]. BPD is a common personality disorder that co-occurs with bipolar disorder. Bipolar disorder and BPD share many common characteristics, and the most crucial overlapping feature is mood instability [5]. About 20% of bipolar II patients and 10% of bipolar I patients have comorbid BPD, and there is a robust relationship between BPD and bipolar disorder II [6].

The presence of comorbid BPD had a negative impact on the clinical course of bipolar disorder as the patients had an unfavorable illness trajectory, higher likelihood of hospitalization, longer treatment duration and worse response to treatment [7]. Riemann et al. concluded from a study in a multicenter outpatient that bipolar patients with BPD had a higher treatment drop-out rate, increased risk of substance abuse, and greater impairment of social and occupational functioning [8]. As bipolar disorder and BPD are each risk factors for suicidal behavior, patients with co-occurring bipolar disorder and BPD present with a higher suicide risk [9].

The quality of life and disease outcomes in bipolar patients are adversely affected by the presence of comorbid BPD [7,8]. Given the increased risk of psychiatric hospitalizations and suicide [9], the assessment and treatment of comorbid BPD in bipolar patients is crucial for improved patient outcomes and reducing suicide risk. Also, many studies in the past found significant linkage between bipolar disorder and BPD [10,11,12], whereas others did not consider BPD’s inclusion on the bipolar spectrum [6,13,14,15]. To our knowledge, no studies have been conducted to address the prevalence and impact of comorbid BPD in bipolar disorder patients during inpatient psychiatric hospitalization. The primary objective of our study is to assess and compare the demographic and clinical characteristics of bipolar patients with versus without BPD and determine the impact of comorbid BPD on the clinical outcomes and health care utilization in an inpatient setting.

## 2. Methods

### 2.1. Data Source

We conducted a retrospective analysis over a five-year period using the Healthcare Cost and Utilization Project’s (HCUP) Nationwide Inpatient Sample (NIS) data from January 2010 to December 2014 [16]. The HCUP database determines the patterns in comorbidities, healthcare utilization and cost across the US, and is sponsored by the Agency for Healthcare Research and Quality (AHRQ). This database represents many non-federal community hospitals and is the largest inpatient database in the US.

### 2.2. Case and Control Selection

We analyzed the NIS database to select the patients between 18 and 75 years. Based on the validated International Classification of Diseases, 9th Revision, and Clinical Modification (ICD-9-CM) diagnosis codes, the patients with a primary diagnosis (DX1) of bipolar disorder and a comorbid diagnosis (DX2 to DX25) of BPD were identified as the cases. This patient cohort was compared with the control cohort which included patients with a primary diagnosis (DX1) of bipolar disorder only. In HCUP databases, more than 14,000 ICD-9-CM diagnosis codes are mentioned [16]. Bipolar disorder was identified using ICD-9-CM diagnosis codes 296.40, 296.41, 296.42, 296.43, 296.44, 296.50, 296.51, 296.52, 296.53, 296.54, 296.60, 296.61, 296.62, 296.63, 296.64, and 296.7; and BPD was identified using ICD-9-CM diagnosis code 301.83. We also performed a retrospective cohort analysis to derive a control cohort that had similar demographic characteristics to the cases. The controls were matched for age, gender and race using the case-control matching application in SPSS version 23 (IBM Corp., Armonk, NY, USA). Finally, a separate cohort was extracted from the NIS of patients (18–75 years) with primary diagnoses of BPD to compare their sociodemographic characteristics and hospital outcomes with bipolar patients with and without BPD.

### 2.3. Variables of Interest

The demographic variables examined in this study included age group (18–35, 36–55, and 56–75 years), gender (male or female), and race (Caucasian, African American, Hispanic, Asian, Native American, and other).

To measure the differences in hospitalization outcomes in bipolar patients with versus without BPD, the outcome variables of interest included the severity of illness that measures the loss of function and disposition of the patient [17]. The All Patient Refined Diagnosis Related Groups (APR-DRGs) are assigned using software made by 3M-Health Information Systems. This severity measure includes the APR-DRG and severity of illness subclass mentioned as a minor, moderate or major loss of function [17]. We calculated the inpatient length of stay (LOS) as the number of nights the patient remained in hospital for the primary diagnosis (DX1). The total charges during hospitalization include emergency department charges but do not include professional fees or non-covered charges [17].

Suicide and intentional self-inflicted injury as the other diagnoses within the patient’s records were identified using the Clinical Classification Software diagnosis code 662 [18]. This included ICD-9-CM diagnosis codes E9500–E9511, E9518, E9520, E9521, E9528–E9531, E9538, E9539, E954, E9550–E9559, E956, E9570–E9572, E9579–E9589, E959, and V6284. The utilization of electroconvulsive treatment (ECT) during hospitalization was identified using ICD-9-CM procedure code 94.27.

Comorbidities are considered coexisting conditions to bipolar disorder, which is the primary diagnosis under our study. AHRQ comorbidity software was utilized to generate binary variables [14]. Using ICD-9-CM codes, these variables identified two comorbidities in the records of discharge: alcohol abuse (291.0–291.3, 291.5, 291.8, 291.81, 281.82, 291.89, 291.9, 303.00–303.93, 305.00–305.03) and drug abuse (292.0, 292.82–292.89, 292.9, 304.00–304.93, 305.20–305.93, 648.30–648.34).

### 2.4. Approaches

Summarization of the results was done using descriptive statistics and cross-tabulation. Continuous variables were explained by using mean and standard deviation (SD). In order to denote categorical data and continuous data, we used a two-sided Pearson’s Chi-squared test and a two-tailed Independent Sample *t*-test, respectively. A multinomial logistic regression model was used to measure the likelihood of associations using odds ratios (OR), *p*-value and 95% Confidence Interval (95% CI) in terms of LOS, total charges, severity of illness, comorbidities, suicidal ideations, and utilization of ECT. Discharge weights within the NIS database [19] were applied in all regression models to obtain a nationally representative inpatient sample. Age, race, gender and median household income of the patient were adjusted. A *p*-value <0.001 was chosen to be the reference value to measure statistical significance. SPSS version 23 (IBM Corp., Armonk, NY, USA) was used to do all the statistical analysis in this study.

### 2.5. Ethical Approval

To maintain confidentiality and the privacy of the patients, physicians, and hospitals, the NIS database de-identifies the state and hospital identifiers using KEY_ID. Permission from the Institutional Review Board was not required for this study as NIS data does not contain patient identification.

## 3. Results

### 3.1. Demographic Characteristics

This study analyzed 510,611 hospital admissions for bipolar disorder that included 268,232 cases with comorbid BPD and 242,379 matched-controls (without BPD). A higher proportion of comorbid BPD was seen in young adults (18–35 years, 53.9%). Comorbid BPD was seen in lower proportions in bipolar patients aged above 55 (6.9%). The majority of the inpatients with bipolar disorder and comorbid BPD were female (84.2%) and Caucasian (83.1%) as shown in Table 1. These disorders were more prevalent in patients from lower socioeconomic status as more than half of the bipolar patients with BPD (N = 152,747, 58.6%) had a median household income <50th percentile. The demographic distribution of bipolar disorder, bipolar disorder with BPD and BPD only is shown in Table 1.

### 3.2. Differences in Inpatient Outcomes

There was no statistically significant difference between bipolar patients with BPD (6%) and without BPD (5.5%) in terms of major severity of illness (OR = 1.047; 95% CI 1.011–1.084; *p* = 0.009). Additionally, comorbid substance use disorder, alcohol abuse, and drug abuse had no significant difference in prevalence in both groups. Suicidal ideation was reported in a higher proportion of bipolar patients with BPD (44.7%) as compared to 43.7% of the BPD only patients and 30% of the bipolar patients without BPD (*p* < 0.001). Therefore, the risk of suicidality was higher in bipolar patients with BPD compared to the controls (OR = 1.418; 95% CI 1.384–1.454; *p* < 0.001).

The mean inpatient LOS per admission was higher in bipolar patients with BPD as compared to controls (6.62 days vs. 5.58 days, *p* < 0.001). The median LOS for all bipolar patients was four days. Bipolar patients with BPD had higher odds of longer hospitalization above the median four days LOS than controls (OR = 1.283; 95% CI 1.251–1.315; *p* < 0.001). The mean total charges were also higher in bipolar patients with BPD ($17,424 vs. $14,527, *p* < 0.001). The median total charges for the study population was $10,287. About 53.3% bipolar patients with BPD had higher odds of total charges greater than the median (OR = 1.239; 95% CI 1.1209–1.270; *p* < 0.001) as compared to 45.6% patients without BPD. The differences in inpatient outcomes are shown in Table 2.

Approximately 2.3% of the bipolar patients with BPD were treated with ECT as compared to 1.5% of the patients without BPD (*p* < 0.001). Consequently, bipolar patients with BPD had a greater likelihood of utilizing ECT as an integral part of psychiatric management (OR = 1.442; 95% CI 1.373–1.515; *p* < 0.001) compared to patients without BPD. The key inpatient outcomes of bipolar patients with BPD compared with the controls as the reference category in the regression model are shown in Table 3.

### 3.3. Predictors of Adverse Inpatient Outcomes in Bipolar Patients with BPD

Older bipolar patients (55–75 years) had 2.5-fold higher odds of LOS of more than four days (OR = 2.523; 95% CI 2.378–2.677; *p* < 0.001) and total charges of more than $10,287 (OR = 2.503; 95% CI 2.359–2.655; *p* < 0.001). Bipolar patients with major severity of illness had a higher likelihood of longer hospitalization (OR = 1.734; 95% CI 1.646–1.827; *p* < 0.001) and higher total charges during hospitalization (OR = 1.873; 95% CI 1.776–1.976; *p* < 0.001).

Patients receiving ECT as part of inpatient management of bipolar disorder had about a five-fold higher likelihood of longer hospitalization (OR = 4.967; 95% CI 4.500–5.483; *p* < 0.001) and an eight-fold greater likelihood of total charges more than the median (OR = 8.105; 95% CI 7.168–9.164; *p* < 0.001). Bipolar males with BPD had higher odds of suicidality than females (OR = 1.126; 95% CI 1.102–1.151; *p* < 0.001). Also, bipolar patients with major severity of illness were more likely to be suicidal (OR = 1.484; 95% CI 1.412–1.558; *p* < 0.001). The predictors of adverse outcomes of bipolar patients with BPD are mentioned in Table 4.

## 4. Discussion

This analysis of nationwide inpatient data is a first attempt to understand the demographic and clinical characteristics of inpatients with bipolar disorder and BPD and assess the impact of comorbid BPD on health care utilization and patient outcomes.

Our study findings of female preponderance (84.2%) in the bipolar with BPD group corroborate the study findings reported by Fornaro et al. [7] which concluded a lower prevalence of comorbidity of bipolar disorder and BPD in males. Another study, by Patel et al., found a higher prevalence of personality disorders in bipolar females (20.8%) with 1.66-fold higher odds than 13.5% males [20]. Contrary to the earlier literature, we noted a higher prevalence of comorbid BPD in young adults (18–35 years). This variation may be explained by the age characteristics of hospitalized bipolar disorder and BPD patients, a population that has never been studied before and may need further studies to replicate our findings.

Riemann et al. reported that bipolar disorder patients with comorbid BPD are more likely to be hospitalized and have an increased risk of substance abuse [8]. As per the National Comorbidity Survey Replication, the prevalence of alcohol abuse and drug abuse in bipolar patients is 39.1% and 28.8% respectively [3]. Our study findings indicate a higher prevalence of drug abuse in bipolar patients with BPD (38.3%) as compared to those without BPD (34.4%). Bipolar patients with BPD in our study had a higher likelihood of longer LOS and higher total charges during hospitalization than the patients without BPD. Our study findings support the earlier evidence reported by Marcinko et al. regarding higher suicide risk in bipolar patients with comorbid BPD [9]. Based on our results, bipolar patients with BPD had 1.4-fold higher odds of suicidality compared to patients without BPD. Bipolar males with BPD were more suicidal than females, and those with severe morbidity had a 1.5-fold higher likelihood of suicidal ideation.

Regarding treatment utilization, our study noted that 2.3% of bipolar patients with comorbid BPD utilized ECT and had a 1.4-fold higher likelihood of receiving inpatient ECT. This may be partly attributed to the higher prevalence of suicidal ideations, and ECT is one of the most effective treatments for the management of suicidal depression [21]. We also noted that the use of ECT was associated with higher healthcare costs due to extended LOS. With recent advances in pharmacogenetic testing (PGT), a study conducted by Ielmini et al. found it to be an effective and individualized therapeutic measure for managing patients with bipolar disorder. About 40% of a patient’s regimen was changed based upon the PGT results with a significant clinical improvement at the three-month follow-up (*p* = 0.001) [22]. There is a need to evaluate if PGT can be efficacious in managing patients with comorbid mental illness like BPD.

In the BPD only cohort, there were 11,007 inpatient admissions with a primary diagnosis of BPD. These patients had a similar LOS as seen in bipolar patients with BPD but lower mean total charges per inpatient admission. Despite a higher proportion of patients with major severity of illness and suicidality, the utilization of ECT was very low (0.6%). This could explain the discrepancy in hospitalization charges. Future studies should be conducted to evaluate the comorbidities that increase the chances of hospitalization in BPD patients.

The main limitation of this study is the lack of patient-level data since NIS is an administrative database. NIS data regarding bipolar disorder and BPD diagnoses are limited to inpatient hospitalization only and do not include data from outpatient settings. Re-hospitalizations, which add to the total inpatient burden, are not accounted for in our study, given the nature of the data. Selection bias is of concern, as this is a retrospective study. In addition, due to the lack of ICD-9-CM codes for psychotropic medications, we could not evaluate other treatment measures if given during inpatient management. However, despite these limitations, NIS is still an excellent population-based representation of disease associations with systematic and temporal factors. However, the main strength of this study is the national representation provided by the dataset, as well as a uniform collection of data obtained over five years through ICD-9-CM codes. Another strength of our study is its large sample size of 510,611 and the reliability of the data, given that the information is coded independently of the individual practitioner and therefore it is subjected to minimal reporting bias. Considering the adverse outcomes, it is clinically imperative to diagnose both bipolar disorder and BPD in patients, and it is also essential to differentiate between both the illnesses [6]. There is still a diagnostic bias for making the final diagnosis in bipolar patients with BPD, and often psychiatrists make the diagnosis that they feel comfortable managing [23]. Psychiatrists need to identify patients’ mood shifts, type of impulsivity, and duration of illness to avoid misdiagnosis of these comorbidities [24,25]. The treatment of bipolar disorder with comorbid BPD can often be challenging due to the lack of evidence-based treatment strategies for optimal management, especially in inpatient settings. The primary treatment for the management of BPD in bipolar patients is psychotherapy [9], which includes psychoanalytic and dialectical behavioral therapies [26]. However, effective psychotropic medication management improves the overall functioning of bipolar patients [9]. In addition, Perugi et al. reported the efficacy of ECT with a favorable clinical outcome in 68.8% of bipolar patients in psychiatric inpatient settings [27].

## 5. Conclusions

Bipolar disorder patients with comorbid BPD tend to be younger Caucasian females. Evaluating younger patients will be a challenge as the early onset of bipolar symptoms is tough to differentiate from BPD [28]. Presence of comorbid BPD in bipolar disorder is associated with a longer LOS, higher charges during hospitalization, increased risk of drug abuse, higher suicide risk, and greater utilization of ECT. Further studies in the inpatient setting are warranted to develop effective clinical strategies for optimal outcomes and reduction of suicide risk in bipolar patients with comorbid BPD.

## Figures and Tables

**Table 1:** Demographic distribution of inpatients with bipolar disorder (BD), bipolar disorder and borderline personality disorder (BD + BPD) and borderline personality disorder (BPD) only.

Variable	BD Only		BD + BPD		BPD Only	
	N	%	N	%	N	%
Age						
18–35 years	129,398	53.4	144,629	53.9	6359	57.8
36–55 years	95,482	39.4	104,975	39.1	4069	37.0
56–75 years	17,499	7.2	18,628	6.9	578	53
Gender						
Male	38,763	16.0	42,505	15.8	1593	14.5
Female	203,616	84.0	225,688	84.2	9414	85.5
Race						
Caucasian	201,451	83.1	199,433	83.1	7041	82.4
Black	17,293	7.1	16,937	7.1	761	8.9
Hispanic	13,675	5.6	13,664	5.7	360	4.2
Asian	2139	0.9	2116	.9	85	1.0
Native American	1351	0.6	1419	.6	77	0.9
Other	6469	2.7	6381	2.7	218	2.6
Median Household Income						
0–25th percentile	58,238	24.8	79,208	30.4	3079	29.0
26th–50th percentile	60,820	25.9	73,539	28.2	3280	30.9
51st–75th percentile	63,153	26.9	60,608	23.2	2832	26.7
76th–100th percentile	52,582	22.4	47,381	18.2	1411	13.3

**Table 2:** Inpatient outcomes of bipolar disorder patients.

Variable	BD Only		BD + BPD		BPD Only		*p* Value
	N	%	N	%	N	%	
Severity of illness							
Minor	83,547	34.5	98,341	36.7	3786	34.4	<0.001
Moderate	145,496	60.0	153,781	57.3	6521	59.2	
Major	13,336	5.5	16,111	6.0	700	6.4	
Comorbid substance use							
Alcohol abuse	53,693	22.2	57,290	21.4	2246	20.4	<0.001
Drug abuse	83,276	34.4	102,342	38.2	4164	37.8	<0.001
LOS per admission, in days							
Mean (±SD)	5.58 (±6.04)		6.62 (±8.33)		6.23 (±9.76)		<0.001
>4 (median)	109,869	45.3	144,447	53.9	5114	46.5	<0.001
Total charges per admission, in USD							
Mean (±SD)	14,527 (±19,159)		17,424 (±26,152)		15,140 (±21,170)		<0.001
>10,287 (median)	110,638	45.6	142,913	53.3	5186	47.1	<0.001
Suicidality							
Suicidal ideation	72,823	30.0	119,997	44.7	4815	43.7	<0.001
Utilization of ECT							
ECT	3691	1.5	6059	2.3	63	0.6	<0.001

Significant *p*-values ≤ 0.001 at 95% Confidence Interval for the comparison between proportions of BD only and BD + BPD groups. Proportions between groups were evaluated using cross tabs and a Chi-squared test and the continuous variables (length of stay and total charges) were evaluated using an Independent Sample *t*-test. Median value in the length of stay and total charges refers to the median value of the total sample. BD: bipolar disorder; BPD: borderline personality disorder; SD: standard deviation; LOS: length of stay; ECT: Electroconvulsive treatment; USD: United States Dollars.

**Table 3:** Outcomes of inpatients with bipolar disorder and borderline personality disorder.

Variable	OR	95% CI	*p* Value
Major severity of illness	1.047	1.011–1.084	0.009
Comorbid alcohol abuse	0.955	0.937–0.974	<0.001
Comorbid drug abuse	1.077	1.058–1.097	<0.001
LOS > 4 days	1.283	1.251–1.315	<0.001
Total charge > $10,287	1.239	1.209–1.270	<0.001
Suicidal ideation	1.418	1.384–1.454	<0.001
Utilization of ECT	1.442	1.373–1.515	<0.001

Significant *p*-values ≤ 0.001 at 95% Confidence Interval. Odds Ratio generated by logistic regression model were adjusted for age, gender and race. Reference category in the model is bipolar disorder only group. OR: odds ratio; CI: Confidence Interval; LOS: length of stay; ECT: Electroconvulsive treatment.

**Table 4:** Predictors of Adverse Outcomes for Inpatients with Bipolar Disorder and Borderline Personality Disorder (BD + BPD).

Variable	OR	95% CI	*p* Value
Length of stay >4 days (median)			
Older age, 55–75 years	2.523	2.378–2.677	<0.001
Major severity of illness	1.734	1.646–1.827	<0.001
Utilization of ECT	4.967	4.500–5.483	<0.001
Total charge >$10,287 (median)			
Older age, 55–75 years	2.503	2.359–2.655	<0.001
Hispanic	1.151	1.083–1.223	<0.001
Major severity of illness	1.873	1.776–1.976	<0.001
Utilization of ECT	8.105	7.168–9.164	<0.001
Suicidal ideation			
Male	1.126	1.102–1.151	<0.001
Major severity of illness	1.484	1.412–1.558	<0.001

Significant *p*-values ≤ 0.001 at 95% Confidence Interval. Odds Ratio generated by logistic regression model were adjusted for age, gender and race. Reference category in the model is bipolar disorder only group. OR: odds ratio; CI: Confidence Interval; LOS: length of stay; ECT: Electroconvulsive treatment.
